# Future directions in ventilator-induced lung injury associated cognitive impairment: a new sight

**DOI:** 10.3389/fphys.2023.1308252

**Published:** 2023-12-18

**Authors:** Yinuo Liu, Xintong Cai, Ruiying Fang, Shengliang Peng, Wei Luo, Xiaohong Du

**Affiliations:** ^1^ Department of Anesthesiology, The Second Affiliated Hospital of Nanchang University, Nanchang, China; ^2^ The Clinical Medical College of Nanchang University, Nanchang, China; ^3^ Department of Sports Medicine, Huashan Hospital, Fudan University, Shanghai, China

**Keywords:** ventilator-induced lung injury, cognitive impairment, inflammation, hippocampus, LPVS

## Abstract

Mechanical ventilation is a widely used short-term life support technique, but an accompanying adverse consequence can be pulmonary damage which is called ventilator-induced lung injury (VILI). Mechanical ventilation can potentially affect the central nervous system and lead to long-term cognitive impairment. In recent years, many studies revealed that VILI, as a common lung injury, may be involved in the central pathogenesis of cognitive impairment by inducing hypoxia, inflammation, and changes in neural pathways. In addition, VILI has received attention in affecting the treatment of cognitive impairment and provides new insights into individualized therapy. The combination of lung protective ventilation and drug therapy can overcome the inevitable problems of poor prognosis from a new perspective. In this review, we summarized VILI and non-VILI factors as risk factors for cognitive impairment and concluded the latest mechanisms. Moreover, we retrospectively explored the role of improving VILI in cognitive impairment treatment. This work contributes to a better understanding of the pathogenesis of VILI-induced cognitive impairment and may provide future direction for the treatment and prognosis of cognitive impairment.

## 1 Introduction

The term ‘ventilator-induced lung injury (VILI)’ refers to pulmonary damage as an adverse consequence of mechanical ventilation characterized by an infiltration of inflammatory-cell and the release of cytokines, which can be classified into four mechanisms: barotrauma (high transpulmonary pressure-mediated lung injury), volutrauma (overdistention-mediated lung injury), atelectrauma (repeated collapse and reopening of alveoli at low lung volumes), and biotrauma (inflammatory response to mechanical stretch) (Slutsky, 1999; Gattinoni et al., 2010; Slutsky and Ranieri, 2013; Beitler et al., 2016a; Curley et al., 2016; Chen L. et al., 2018; Katira, 2019). Cognitive impairment which is generally considered multifactorial is the term used to denote deficits in cognitive domain and behavioral domain which manifest neuroinflammation and structural alterations in the brain (Basso et al., 2007; Sachdev et al., 2014; Perna et al., 2015; de Azevedo et al., 2017; Park, 2017; Alam et al., 2018; Gilmore-Bykovskyi et al., 2018; Rengel et al., 2019; McCollum and Karlawish, 2020; Giordano et al., 2021). Memory impairments, executive impairments, memory plus executive functions impairments, and cognitive rigidity-associated impairments are the four unique profiles of cognitive impairment based on the impairment of memory and/or executive functions (Yu and Lee, 2018).

Injuries due to mechanical ventilation can occur not only in the lungs but also in the brain, pointing to a potential link between VILI and cognitive impairment (Pelosi and Rocco, 2011; Chen et al., 2016). Studies have shown that VILI can stimulate the vagal nerve via inflammatory cytokines such as interleukin (IL)-1β, IL-6, and tumor necrosis factor (TNF)-α which cause hippocampal inflammation (Chen C. et al., 2015; Witzenrath and Kuebler, 2021). Vagal signaling in VILI also can activate type 2 dopamine receptors to trigger hippocampal apoptosis (González-López et al., 2013). In a recent study, a correlation between VILI and neurotransmitter hippocampal imbalances has been proven (Wei et al., 2022). Furthermore, hypoxia as a possible indication of mechanical ventilation inclines the hippocampus to develop atrophy (Borsellino et al., 2016). Another finding is that the mitochondrial apoptosis pathway may be the possible connection between VILI and cognitive impairment (González-López et al., 2019). Of note, VILI may bring about cognitive impairment by blood-brain barrier (BBB) dysfunction and cerebral accumulation of soluble Aβ-1-40 (Lahiri et al., 2019).

Thus far, previous studies have indicated that VILI can contribute to cognitive impairment (Sakusic and Rabinstein, 2018; Rengel et al., 2019; Sasannejad et al., 2019; Sparrow et al., 2021). Nearly one-third of mechanically ventilated patients have impaired performance on neurocognitive testing at 6 months which demonstrates that cognitive impairment is common among patients with VILI (Jackson et al., 2003; Patel et al., 2023). However, it is difficult to disentangle specific complex biomolecular mechanisms to explain the underlying relationship between lung injury and cognitive impairment. Considerable social and family costs burden, damaged emotional health of caregivers, impaired social and occupational competence and many other hazards have been found in discharged patients who have been mechanically ventilated (Jönsson et al., 1999; Bédard et al., 2000; Jackson et al., 2003; Freedman et al., 2022). Therefore, cognitive impairment due to VILI is a growing public health concern that needs to be explored. The main issues addressed in this paper are the implications of VILI for cognitive function and strategies for its treatment and prevention.

## 2 Risk factors

Systematic studies have shown that the incidence of postoperative cognitive dysfunction (POCD) ranges from 17% to 56% shortly after surgery, and that 4%–62% of intensive care unit (ICU) survivors develop extensive cognitive impairment after 2–156 months of follow-up (Wolters et al., 2013; Czyż-Szypenbejl et al., 2019). The high probability of cognitive impairment is strongly associated with the inevitable mechanical ventilation during surgery or ICU treatment (Chen C. et al., 2015; Bassi et al., 2021; Yao et al., 2021). A study showed that VILI induced by mechanical ventilation causes potentially reversible neuronal damage and inflammation in the frontal cortex and hippocampus and leads to delirium (Sparrow et al., 2021), which indicates us that VILI is included as a risk factor of cognitive impairment. One of the main causes of VILI is the setting of various parameters during ventilation (Marini et al., 2023), such as too high or too low oxygen or carbon dioxide concentration, which may cause certain damage to the brain, as we mentioned in Table 1. Fortunately, there is evidence that mechanical ventilation combined with interventions, such as minimizing the tidal volume of mechanical ventilation, can help significantly reduce the duration of delirium and help prevent the occurrence of long-term cognitive impairment (Sasannejad et al., 2019). Here we describe the VILI-related factors that induce cognitive impairment, in order to further improve the prevention of related factors, as well as strengthen the relevant preventive measures (Table 1).

**TABLE 1:** Risk factors of cognitive impairment related to VILI.

Risk factors	Experimental studies	Clinical studies	Refs
Hypoxia-exposure	Hypoxia is an important factor for hippocampal microglia to produce inflammatory factors, which may impair cognitive function	MV may affect cerebral oxygen saturation, whose reduction may predict POCD in elderly patients with total knee arthroplasty	Ni et al. (2015), Ding et al. (2018)
Hyperoxia-exposure	When rats are subjected to high oxygen oxidative stress, the accumulation of lipid peroxidation products in the cerebral cortex and hippocampus leads to significant loss of learning and memory function	Every 10%·hour of intraoperative hyperoxic cerebral reperfusion was independently associated with a 65% increase in the odds of delirium	Arendash et al. (2009), Lopez et al. (2017)
Hypocapnia	Hypocapnia during anesthesia causes tissue damage in the caudoputamen, which may be responsible for long-lasting postoperative delirium in patients with stroke and/or dementia	Duration and severity of intraoperative hypocapnia were independent risk factors for POCD.	Miyamoto et al. (2001), Mutch et al. (2018)
Hypercapnia	Hypercapnia can significantly increase the expression of NLRP3, caspase-1 and IL-1β in hypoxic-activated hippocampal microglia, which may participate in the pathogenesis of cognitive impairment	Permissive hypercapnia but not severe hypercapnia has a protective effect on postoperative cognitive function by improving cerebral oxygenation	Ding et al. (2018), Li et al. (2023)
Prolonged MV	After exposure to 6h MV, significant changes in synaptic morphology and inflammation level of the hippocampus aggravated memory loss after surgery	Clinically, an increase in the duration of MV may be associated with an increased risk of delirium during hospitalization	Chen et al. (2015a), Bassi et al. (2021)
HVT	MV using HVT for short periods of up to 3.5 h has been shown to induce hippocampal apoptosis	MV with HVT induces intracerebral inflammation and failure	Pinheiro de Oliveira et al. (2010), Lellouche et al. (2012), Rohrs et al. (2020)
High PEEP	With the increase of PEEP, ICP increased, and MAP and intracranial compliance decreased	PEEP up to 20 cm H2O significantly reduced MAP and cerebral blood flow, which may impair cognitive function compound effects from other potential mechanisms	Georgiadis et al. (2001), Muench et al. (2005), Sasannejad et al. (2019)
ARDS	The cognitive function of pigs in the ARDS group induced by MV was more severely impaired than that in the hypoxia group, and hippocampal inflammation tended to increase	In a study of 55 ARDS survivors, at hospital discharge, 100% of the survivors showed cognitive and emotional impairments, as well as health conditions that affected their quality of life	Hopkins et al. (1999), Bickenbach et al. (2011)

Abbreviation: MV, mechanical ventilation; POCD, postoperative cognitive dysfunction; HVT, high tidal volume; NLPR, NOD-like receptor protein; IL, interleukin; ICU, intensive care unit; SpO2.arterial oxyhemoglobin saturation; PEEP, positive end-expiratory pressure; ICP, intracranial pressure; MAP, mean arterial blood pressure; ARDS, acute respiratory distress syndrome.

## 3 Pathogenesis

**FIGURE 1:**
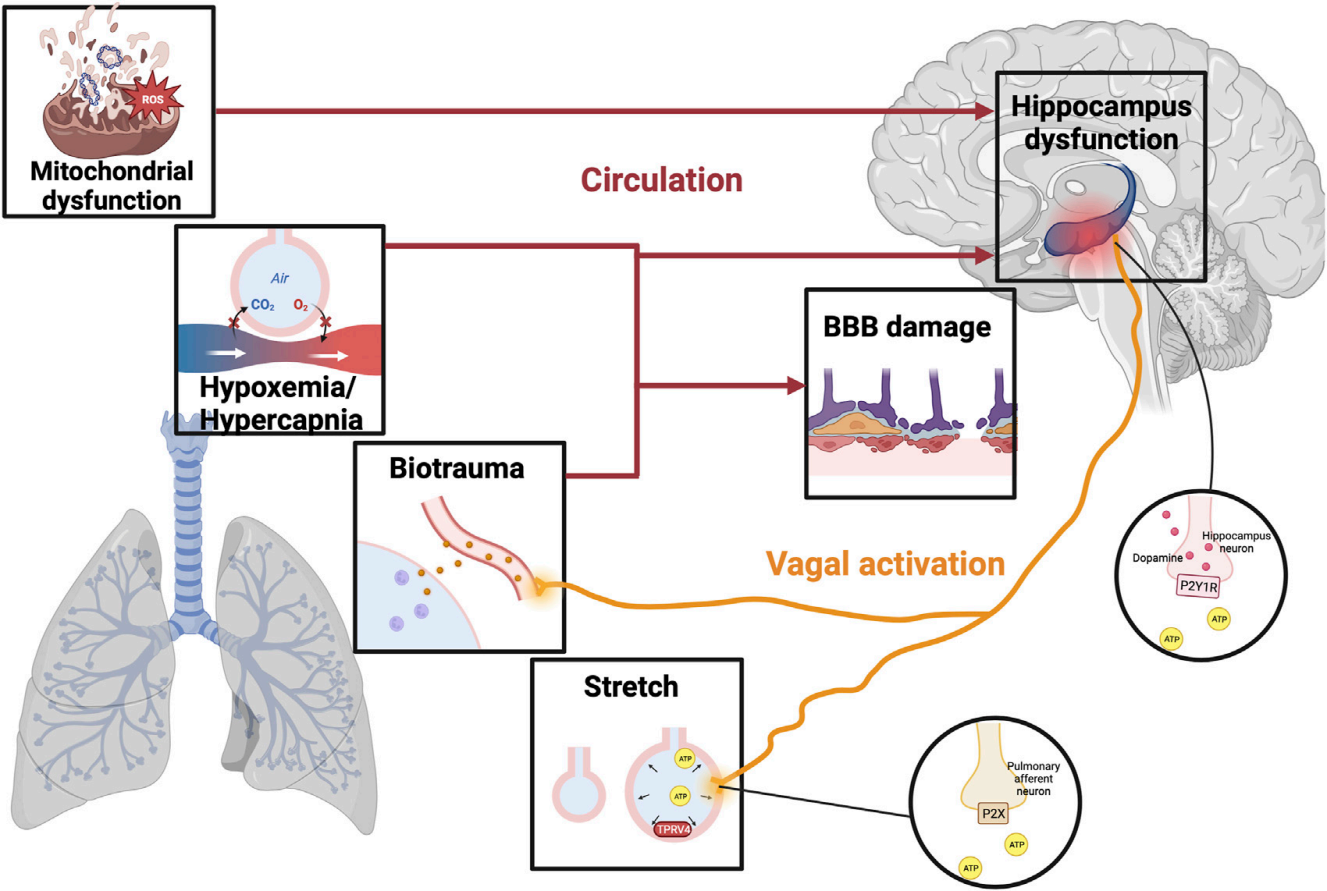
The mechanism of VILI-induced cognitive impairment. Illustrations depict physical alternations related to VILI, including hypoxemia/hypercapnia, mitochondrial dysfunction, biotrauma, and alveolar stretch. Different stimuli can trigger an ascending signal to the brain through direct stimulation of the vagal nerve or circulatory factors, resulting in a breakdown of the BBB and even damage to the hippocampus. In the stretched alveolar, activation of TRPV4 promotes the release of ATP, which activates purinergic receptors on the pulmonary afferent neurons to activate vagal nerve. Moreover, VILI can stimulate hippocampal dopamine release by activating ATP-mediated vagal signaling and P2Y1 receptors, thereby increasing the degree of brain damage leading to cognitive impairment. VILI, ventilator-induced lung injury; TRPV4, transient receptor potential cation channel; ATP, adenosine triphosphate; BBB, blood-brain barrier; P2XR, purinergic P2X ligand-gated ion channel receptor; P2Y1R, purinergic P2Y1 G-protein-coupled receptor.

The impact of lung damage caused by mechanical ventilation can be transmitted to the brain through different pathways. Many experiments have investigated a strong link between the development of cognitive dysfunction after VILI, but the mechanism is still under discussion. We reviewed the relevant literature and further summarized the pathogenesis of cognitive dysfunction in VILI (Figure 1), hoping to stimulate further experimental exploration.

### 3.1 Hypoxemia and hypercapnia

DREYFUSS discovered early changes in the ultrastructure of the blood-gas barrier in VILI model rats (Dreyfuss and Saumon, 1998), and the mechanical injury to the blood-gas barrier is the hallmark of VILI (Biehl et al., 2013). The destruction of the integrity of the gas-blood barrier will undoubtedly affect O2 and CO2 exchange, resulting in cerebral blood flow and metabolism disorders. When oxygen content (CaO2) decreased, the increase of cerebral blood flow (CBF) compensated for the decrease of CaO2 to maintain cerebral oxygen delivery, however, the increase in blood flow does not fully compensate for the lower CaO2 during blood thinning (Hoiland et al., 2016). Previous studies have shown that patients with hypoxemia have more severe cognitive dysfunction than those without hypoxemia (Findley et al., 1986), and evidence shows that reduced cerebrovascular reactivity and/or oxidative stress may play a role (Steinback and Poulin, 2016). More importantly, hypoxia affects the oxidative phosphorylation of nerve cells, and some brain regions, such as the hippocampus, are more susceptible to hypoxic damage and atrophy following hypoxic events (Bilotta et al., 2019). A study of Titus revealed that under the exposure to 7 days of hypoxia, significant atrophy occurs in hippocampal CA1 and CA3 pyramid-neuron dendrites in rats, which is associated with severe impairment of the partially baited radial arm maze task learning (Titus et al., 2007). In addition, a series of evidence suggest that erythropoietin (EPO) can improve cognitive function by protecting hippocampal neurons from hypoxia-induced oxidative damage, further confirming that hypoxia-induced hippocampal damage is associated with long-term brain dysfunction, and these findings are helpful in exploring the crosstalk between acute lung injury and distal brain injury (Dayyat et al., 2012; Jacobs et al., 2021). Besides, excitatory amino acids, energy depletion, and ion-related signal transduction may induce neuronal apoptosis under hypoxia conditions (Banasiak et al., 2000), and the loss of neurons is an important pathological process of cognitive impairment.

Permissive hypercapnia has a protective effect on VILI by reducing lung stretch which is often necessary to improve survival and reduce complications (O'Croinin et al., 2005). Similarly, mild to moderate hypercapnia may have neuroprotective effects after cerebral ischemia, while severe hypercapnia may aggravate neuronal injury (Deng et al., 2020). Ding et al. found that hypercapnia may participate in the increase of IL-1β secretion in hypoxic hippocampal microglia through activation of the NLRP3 inflammasome, and aggravate the apoptosis of hippocampal neurons. Moreover, the overexpression of IL-1β can reduce the expression of cerebral vascular endothelial tight junction protein through the IL-1R1/p-IRAK-1 pathway, further impairing the integrity of the BBB and ultimately exacerbating cognitive dysfunction (Ding et al., 2018; Ding et al., 2020).

### 3.2 Inflammation

The stretching of lung tissue by injurious mechanical ventilation causes VILI which is accompanied by the production of cytokines, and the biotrauma is not limited to the lung but may also lead to systemic inflammatory response syndrome (SIRS) (Halbertsma et al., 2005). When systemic inflammation occurs, the BBB can undergo both disruptive and non-disruptive changes. The normal physiological response of brain endothelial cells to systemic inflammation is non-disruptive, achieving intracranial signal transduction without altering permeability or cellular transmigration through the BBB, translating systemic inflammatory information into changes in neuronal activity (Galea, 2021). Intraperitoneal injection of lipopolysaccharide (LPS) can downregulate the expression of a secondary transporter of thyroid hormone L-type amino acid transporters (LAT)1 mRNA in BBB (Wittmann et al., 2015). Similarly, other transporters can also be up/downregulated by systemic inflammation to varying degrees, such as plasma membrane monoamine transporter (PMAT) (Wu et al., 2015), insulin, and (Xaio et al., 2001) and amyloid β protein (Aβ) (Jaeger et al., 2009). Moreover, cytokines directly mediate some non-destructive BBB changes. IL-1β and TNF-α are the major cytokines involved in VILI (Halbertsma et al., 2005), and their distribution throughout the body causes activation of brain endothelial and glial cells (Nishioku et al., 2010; Krasnow et al., 2017). An immunostaining study suggests that increased cytokine expression may lead to a rupture of the BBB, leading to cognitive impairment in 8-week-old rats of streptozotocin injection (Geng et al., 2018). In addition to these cytokines, known immune-brain signals include prostaglandin E2 (PGE2). Cao studies have shown that circulating IL-1β promotes the expression of cyclo-oxygenase (COX) in brain endothelial cells, thereby promoting the increase of PGE2 levels and affecting the activity of central neurons (Cao et al., 1996; Matsumura and Kobayashi, 2004). The stimulation of systemic inflammation can also induce cellular transmigration be the aftermath of diapedesis, includes recruitment of leukocytes to the brain parenchyma through the BBB (Varatharaj and Galea, 2017). During this process, the expression of intercellular cell adhesion molecule (ICAM-1), vascular cell adhesion molecule (VCAM-1), and chemokine receptor 2 (CXCR2) in endothelial cells is upregulated (Leow-Dyke et al., 2012; Wu et al., 2020), and pericytes also support the transport of neutrophils in BBB by IL-8 (Pieper et al., 2013). A study has shown that VILI can increase BBB permeability in rats, which may be related to the expression of tight junction protein in brain tissue (Zhang et al., 2023). Interestingly, an immunohistochemical study of brain tissue from fatal sepsis showed reduced or missing levels of tight junction molecules occludin, zonula occludens-1 (ZO-1), and claudin-5 (Erikson et al., 2020), which indicates that systemic inflammation is related to the breakdown of tight junctions, thus explaining the leakage of solutes across the BBB. Besides, the components of the BBB can also undergo structural changes induced by inflammation (Galea, 2021), and a recent review suggests that BBB breakdown is an early biomarker of human cognitive dysfunction (Nation et al., 2019).

Similarly, VILI can also induce inflammation in the brain parenchyma, which may lead to synaptic remodeling and neurodegeneration within the hypothalamus, altering hypothalamus-associated nerve conduction, and ultimately leading to disruption of cognitive function mediated by regions such as the hippocampus and amygdala (Miller and Spencer, 2014). An experiment by Chen et al. found that the cognitive ability of mice subjected to mechanical ventilation for 6 h was significantly decreased, and it was speculated that this may be related to the destruction of hippocampal structure and the increase of hippocampal IL-1β, IL-6, and TNF-α levels (Chen C. et al., 2015). In addition, a recent experiment found that the use of IL-6 inhibitors in VILI model mice significantly reduced frontal cortex and hippocampus inflammation, thereby improving cognitive impairment (Sparrow et al., 2021). Currently, there have been several experiments on protecting neurons by suppressing inflammation to improve cognitive impairment. For instance, Belarbi’s data suggest that the TNF-α synthesis inhibitor 3,6′-dithiothalidomide can significantly reverse hippocampal-dependent cognitive deficits induced by chronic neuroinflammation (Belarbi et al., 2012).

### 3.3 Neural pathway

There exists a direct neural pathway that connects different regions of the lung and brain stem to the hippocampus to transmit mechanical ventilation stimulation. Positive airway pressure has been shown to activate stretching and chemical receptors in the airways and lung stroma, and different stimuli can trigger upward signals reaching the brain via direct neural connections or circulatory factors (Albaiceta et al., 2021). A study by González-López showed that stretch/strain alternations stimulate transient receptor potential vanilloid (TRPV)4 channels to release adenosine triphosphate (ATP) and activate purinergic P2X ligand-gated ion channel receptor (P2XR) present on the pulmonary afferent neurons, thereby stimulating vagal signals to cause ventilator-induced brain injury (González-López et al., 2019). Besides, VILI can induce hippocampal inflammation by stimulating the vagal nerve with inflammatory cytokines (González-López et al., 2013; Chen C. et al., 2015), and when Zielinski et al. severed the vagal nerve in mice, brain damage induced by TNF-α and LPS-induced brain cell factors in mouse peripheral blood was improved (Zielinski et al., 2013).

Recent studies have found a correlation between VILI and hippocampal neurotransmitter imbalances, however, few studies have been presented so far, so only dopamine will be focused as follows. Dopamine acts as a key neurotransmitter in the brain that mediates movement, cognitive function, learning, and memory function. Dopamine, as an anti-inflammatory substance, can significantly reduce lung tissue damage in rats by inhibiting the NLRP3 signaling pathway, which is a potentially effective treatment strategy for VILI (Yang et al., 2017). Moreover, dopamine improves alveolar fluid reabsorption in high tidal volume (HVT)-ventilated rats by up-regulating Na^+^-K^+^-ATPase function in alveolar epithelial cells (Saldías et al., 2002). However, mechanical ventilation promotes the release of dopamine by causing increased expression of ATP and purinergic P2Y1 G-protein-coupled receptor (P2Y1R) in stretched lung epithelial cells to exacerbate brain injury (Wei et al., 2022). In addition, mechanical ventilation activates the type 2 dopamine receptors to block the pro-survival Akt/GSK3b pathway and thus activates the hippocampal apoptotic cascade *in vivo* and *in vitro* experiments (González-López et al., 2013). Therefore, the use of dopamine in mechanical ventilation requires weighing up the pros and cons, which is subject to further evaluation by clinicians.

### 3.4 Mitochondria

Mitochondrial dysfunction is an important part of VILI development. Excessive mechanical ventilation with HTV triggers mitochondrial damage and autophagy leading to the release of mitochondrial DNA (mtDNA), which is thought to be the damage-associated molecular patterns (DAMPs) (Lin et al., 2018). Circulating mitochondrial DAMPs are major players in systemic inflammatory responses by stimulating neutrophils (Zhang et al., 2010). Besides, a study reported the synapses in hippocampal CA1 region of mice ventilated for 6 h showed conspicuous mitochondrial swelling and vacuolation under transmission electron microscopy (Chen C. et al., 2015). Persistent mitochondrial function impairment in ventilation results in no net time for key recovery processes such as autophagy, mitophagy, or mitochondrial biogenesis to activate, thereby reducing brain ATP levels and oxygen consumption leading to cognitive impairment (Manfredini et al., 2019). Injured mitochondria are also associated with increased production of reactive oxygen species (ROS). Excessive ROS in VILI results in activation of the NLRP3 inflammasome and plays a key role in cognition decline by elevating brain inflammation (Chen L. et al., 2015; Parker, 2018). Wei’s experiment confirmed that ROS-induced NLRP3 activation from mitochondria is a key upstream mechanism controlling IL-1β secretion in hippocampal microglia, and this pathway mediates neuroinflammation involved in the occurrence and development of POCD (Wei et al., 2019). Moreover, evidence from Boscolo et al. suggests that ROS upregulation may also lead to impairment of cognitive function due to membrane lipid peroxidation and neuron loss (Boscolo et al., 2012). Therefore, the current treatment of mitochondrial dysfunction has become an important means of cognitive disorders.

## 4 Prevention and treatment strategies

As mentioned above, mechanical ventilation can cause lung damage, i.e., ventilator-induced lung injury from various aspects such as inflammatory response and neurotransmitter alterations, but it has been shown that appropriate mechanical ventilation can alter clinical outcomes in mechanically ventilated patients (Brower et al., 2000). Not coincidentally, this double-edged role also exists in pharmacological interventions. Preventive and therapeutic strategies that may improve cognitive impairment due to VILI will be explored below from different perspectives.

### 4.1 Ventilatory strategies

Apart from Beitler et al. who suggested in a cohort study that lower tidal volumes after out-of-hospital cardiac arrest are associated with good cognitive function (Beitler et al., 2017), there are no clinical studies for the time being that report on the preventive and therapeutic efficacy of different modes of ventilation in the prevention of cognitive impairment due to VILI. Therefore, in terms of ventilation modes, this paper will focus on the research progress in treating VILI. Various ventilation modes such as lung protective ventilation strategies (LPVS), open lung approach, and high-frequency oscillatory ventilation have been intensively studied, and these ventilation modes will be analyzed and compared in the following.

#### 4.1.1 Lung protective ventilation strategies (LPVS)

Depending on the differences in therapeutic aims and approaches, lung protective ventilation strategies can be broadly categorized into low tidal volume (LVT) ventilation and positive end-expiratory pressure (PEEP). In terms of the mode of ventilation used to provide an LPVS, pressure-controlled ventilation is now increasingly being used by clinicians as an LPVS as opposed to volume-controlled ventilation, which has traditionally been more commonly used (Prella et al., 2002). This is because the variable and decelerating nature of the inspiratory flow pattern of pressure-controlled ventilation recruits the alveoli for a longer period of time and prevents alveolar hyperinflation through a more even distribution of gas (Rose, 2010).

LVT ventilation reduces VILI by reducing lung hyperextension and the release of inflammatory mediators, whereas PEEP’s means of ameliorating lung injury may be by inducing alveolar recruitment to avoid repeated airway collapse and opening (Brower et al., 2000; Crotti et al., 2001). A systematic review investigating LVT ventilation in patients with respiratory distress syndrome and acute lung injury found evidence that a ventilation strategy using a tidal volume equal to or less than 7 mL/kg of measured body weight and plateau pressure less than 31 mm H2O reduced 28-day mortality and concluded that lower tidal volume ventilation reduces the relative risk of death at day 28 and hospital mortality (Petrucci and De Feo, 2013). A multicenter randomized controlled trial of 767 adults comparing an LVT plus high PEEP strategy (28–30 cm H2O) with an LVT plus medium PEEP strategy (5–9 cm H2O) found that high PEEP aimed at limiting hyperinflation and increasing alveolar recruitment improved lung function and reduced the duration of mechanical ventilation and organ failure (Mercat et al., 2008). In a randomized controlled clinical trial related to both of the above, it was found that a group with an LVT (5–8 mL/kg of predicted body weight) and PEEP set on day 1 at the lower inflection point on the pressure-volume curve of the respiratory system ( $P_{flex}$ ) plus 2 cm H2O has a more significant improvement in ICU mortality than a group with a large tidal volume (9–11 mL/kg of predicted body weight) and a relatively low PEEP (Villar et al., 2006).

However, an LVT ventilation strategy may result in the need for patients to receive larger doses of sedation, a practice independently associated with coma and delirium (Seymour et al., 2012). It has also been suggested that elevated PEEP may increase dynamic and end-inspiratory lung stress if it is not effective in inducing alveolar recruitment, which may exacerbate lung inflammation and injury (Sahetya et al., 2017). From these perspectives, the use of LPVS to treat cognitive impairment caused by VILI needs to be further investigated and improved.

#### 4.1.2 Open lung approach

The open lung approach achieves better levels of pulmonary ventilation in patients primarily through recruitment maneuvers followed by higher levels of PEEP (Lachmann, 1992). Recruitment resuscitation increases pulmonary airway pressure to reverse atelectasis, and higher levels of PEEP keep the alveoli open (Sahetya and Brower, 2017). Recruitment maneuvers can be achieved by using pressure-controlled ventilation after hemodynamic stabilization and the most reported method is a continuous positive airway pressure mode with a Paw of 35–45 cm H2O applied for 30–40 s (Keenan et al., 2014). The optimal setting of the PEEP level required by the patient needs to be determined by PEEP titration which has many approaches (Hess, 2015). A prospective multicenter randomized controlled trial study has demonstrated that the open lung approach can not only be safely applied to patients with acute respiratory distress syndrome (ARDS) but that patients experience significant improvements in oxygenation, lung mechanics, and driving pressures (Kacmarek et al., 2016). However, results from a randomized trial of 1,010 patients showed that recruitment resuscitation and PEEP titration increased the risk of barotrauma and death with barotrauma (Cavalcanti et al., 2017). In addition to this, three large, randomized trials failed to show the benefit of the open lung approach on clinical outcomes (Briel et al., 2010). Therefore, carefully conducted trials are needed to determine whether the open lung approach as an effective treatment for improving symptoms in VILI patients.

#### 4.1.3 High-frequency oscillatory ventilation

High-frequency oscillatory ventilation is considered the ideal form of ventilation with a LVT that may be smaller than the anatomical dead space and a mean airway pressure that is higher than the conventional PEEP, and it has a typical range of frequencies of 4–6 Hz for adults, which can be as high as 15 Hz (Pillow, 2005; Fessler et al., 2008; Herrmann et al., 2020). The mechanisms by which high-frequency oscillatory ventilation reduces lung injury are similar to typical lung-protective ventilation strategies, both of which are associated with preventing alveolar overdistension, avoiding repetitive alveolar collapse and opening, and uniform lung inflation (Papazian et al., 2005; Fessler et al., 2008). A systematic evaluation and meta-analysis of one study showed that high-frequency oscillatory ventilation improves patient oxygenation (Huang CT. et al., 2014). Nevertheless, several other clinical studies, as well as the former, have found that although high-frequency oscillatory ventilation has the potential to ameliorate lung injury, it does not provide a survival benefit and may increase complications and mortality (Young et al., 2013; Huang CT. et al., 2014; Lall et al., 2015; Goligher et al., 2017).

#### 4.1.4 Airway pressure release ventilation (APRV)

Airway pressure release ventilation is a form of continuous positive airway pressure (CPAP) ventilation characterized by intermittent partial pressure release and voluntary breathing independent of the ventilator cycle (Downs and Stock, 1987). Preemptive application of airway pressure release ventilation has been demonstrated experimentally and clinically to alleviate lung injury by inhibiting surfactant inactivation and alveolar edema, and to improve oxygenation and respiratory compliance (Habashi, 2005; Emr et al., 2013; Zhou et al., 2017). It is worth noting that the most frequently studied method of setting airway pressure release ventilation parameters, time-controlled adaptive ventilation (TCAV), was more effective in stabilizing lung tissue and reversing collapsed lung tissue compared with other methods of setting parameters (Nieman et al., 2023). The TCAV program designed by Habashi is an APRV model which features Release Phase (swift periods of partial pressure discharge) between CPAP Phase aimed at avoiding alveolar collapse by prolonging inspiratory time and minimizing expiratory time (Nieman et al., 2020; Janssen et al., 2021). With its capacity to stabilize alveoli that are in the midst of atelectrauma and its ability to securely and continually recruit atelectatic tissue over a sufficient span of time, TCAV could assist in mitigating the progression of VILI, which is known as the VILI vortex (the ongoing shrinkage of the baby lung) (Nieman et al., 2022). Given that Bellani et al. identified that LVT failed to notably decrease mortality in ARDS, it is valuable to compare the pros and cons of TCAV to LVT (Bellani et al., 2016). LVT’s low airway pressure, while preventing over-expansion of the lung tissues, contributes to the VILI vortex and may eventually lead to the development of ARDS (Nieman et al., 2022). As reported by Kollisch-Singule et al., the TCAV mode outperformed LVT ventilation in terms of uniformly reopening and stabilizing alveoli, recruiting the chest wall and minimizing alveolar micro-strain (Kollisch-Singule et al., 2019). Furthermore, a systematic review confirmed the favorable results achieved by TCAV in VILI studies when compared to other mechanical ventilation strategies, while noting that evidence for the impact of TCAV in patients with ARDS is still insufficient (Katzenschlager et al., 2023). In brief, more clinical trials are required in order to validate the ARPV’s effectiveness against VILI, and its capacity to alleviate cognitive impairment due to VILI demands to be investigated more in-depth.

### 4.2 Pharmacologic interventions

No pharmacologic interventions have been shown experimentally or clinically to directly improve cognitive outcomes in mechanically ventilated patients with VILI, but clinical outcomes such as delirium-free and coma-free alive days in some studies may suggest that the pharmacologic interventions studied improve cognitive function in mechanically ventilated patients, as mechanical ventilation is an independent risk factor for delirium, and delirium is an acute disturbance of consciousness and cognition with possible complications of persistent cognitive impairment post-discharge and prolonged mechanical ventilation (Ely et al., 2004; Pandharipande et al., 2013). Parallel to this, the effect of pharmacological interventions on VILI will also be analyzed in this section.

#### 4.2.1 Antipsychotics

Intravenous haloperidol is recommended by The American College of Critical Care Medicine as a temporary measure for the occurrence of agitation in patients with delirium, but not as a preventive and resolution measure (Jacobi et al., 2002). Devlin concludes that the addition of quetiapine to intravenous haloperidol relieves delirium (Devlin and Skrobik, 2011). However, in a double-blind, randomized, placebo-controlled trial of 566 mechanically ventilated patients in the ICU, the duration of delirium and coma in the haloperidol group versus the ziprasidone group was not significantly different from the placebo group outcome (Girard et al., 2018). Another Modifying the INcidence of Delirium (MIND) randomized, placebo-controlled trial reached a similar conclusion to the former: neither haloperidol nor ziprasidone improved live days without delirium or coma (Girard et al., 2010). In conclusion, more evidence is now needed on the potential for antipsychotic medication to treat mechanically ventilated patients with delirium.

#### 4.2.2 Sedation

The acute cognitive impairment manifests as delirium and coma that may result from the use of some sedatives in mechanically ventilated patients (Pandharipande et al., 2006), which is contrary to our therapeutic aims, but for this reason, a discussion on how the type of sedation choices can better minimize the impact on cognitive function is warranted. Establishing mild sedation through protocols and assessment scales reduces delirium and duration of mechanical ventilation (Motta et al., 2016). A systematic review showed that benzodiazepine sedatives such as midazolam and lorazepam increased the duration of mechanical ventilation compared to non-benzodiazepine sedatives such as dexmedetomidine and propofol (Fraser et al., 2013). Another prospective, randomized, double-blind study of mechanically ventilated patients who received a sedative titrated to achieve sedation goals assessed cognition with a validated ICU cognitive test battery showed that dexmedetomidine not only preserved cognitive status but also improved cognitive levels below the normal baseline in the majority of patients compared with propofol (Mirski et al., 2010). Furthermore, dexmedetomidine also made it easier for patients to be aroused and communicate about their pain compared to midazolam and propofol (Jakob et al., 2012). More significantly, when compared to the neurotoxicity of other sedatives for the treatment of cognitive impairment, the cerebroprotective properties of dexmedetomidine, manifested in mechanisms featuring decreasing the release of inflammatory mediators, inhibiting the release of excitatory neurotransmitters to prevent neuronal necrosis, and phosphorylating AKT and up-regulating PI3k expression to refrain apoptosis, have been demonstrated in a number of preclinical studies (Chiu et al., 2011; Tanabe et al., 2014; Shen et al., 2017; Tsivitis et al., 2023). For the aspect of VILI itself, Chen et al. found that dexmedetomidine could decrease lung inflammation in VILI by inhibiting the TLR4/NF-κB signaling pathway driven by 2-adrenoceptors, whilst Zhu et al. discovered that the curative impact of dexmedetomidine on the lungs in VILI occurred in conjunction with activation of the ERK1/2 pathway, and both experimental studies could give theoretical support to the above clinical applications (Chen H. et al., 2018; Zhu et al., 2020). In light of the aforementioned studies, we can tentatively conclude that in the choice of sedation, the non-benzodiazepine dexmedetomidine may be better able to reduce the effects on cognitive function in mechanically ventilated patients.

#### 4.2.3 Neuromuscular blocking agents

The previously mentioned LVT ventilation reduces VILI (Brower et al., 2000), but LVT may induce lung injury through breath-stacking asynchrony resulting in a higher than expected volume of gas (Chanques et al., 2013). Chanques et al. found that neuromuscular blocking agents prevented the higher tidal volume-induced lung injury due to breath-stacking asynchrony (Beitler et al., 2016b), but how to limit the dosage and duration of neuromuscular blockers needs to be explored (Bennett and Hurford, 2011). In a prospective randomized experiment, Forel et al. demonstrated that using neuromuscular blocking drugs early on reduced pulmonary and systemic inflammatory responses by lowering IL-1, IL-6, and IL-8 levels (Forel et al., 2006). As reported by Fanelli et al., neuromuscular blocking agents pancuronium and cisatracurium which block nicotinic acetylcholine receptor α1 in animal and cellular studies exert the previously indicated anti-inflammatory potential that can be therapeutic and protective in VILI (Fanelli et al., 2016). Another meta-analysis of 1,461 randomized controlled trials demonstrated that neuromuscular blocking agents have a positive effect on oxygenation and anti-inflammation and have a lower risk of barotrauma (Ho et al., 2020). However, contradicting these positive results, a multicenter study indicated that mechanically ventilated pneumonia patients who used neuromuscular blockers had a longer duration of mechanical ventilation and a higher 90-day mortality rate compared to non-users (Baek et al., 2022). In addition, the clinical outcome of deep sedation in patients with neuromuscular blockers was more negative than the cognitive outcome associated with the recommended mild sedation mentioned above (Pan et al., 2023). The use of neuromuscular blocking agents in mechanically ventilated patients to prevent VILI remains an area of some controversy, besides, the effects of neuromuscular blocking agents on cognitive function are currently uncertain. Therefore, more high-quality research is needed to provide more definitive guidance.

#### 4.2.4 Anti-inflammatory medications

Several studies have revealed the potential of glucocorticosteroids such as dexamethasone and budesonide for the treatment of VILI, where the pathways by which the different drugs produce their effects are not quite the same, although their positive outcomes are similar (Nin et al., 2006; Hegeman et al., 2013; Ju et al., 2016). Non-steroidal anti-inflammatory medications (NSAIDs), in addition to glucocorticoids, have demonstrated noteworthy beneficial effects against inflammation in animal models of VILI. As reported by Huang et al., the alleviation of VILI by ibuprofen is linked to the suppression of Rho-kinase activity, while Rho-kinase has been revealed to be associated with axonal regeneration, implying the potential of ibuprofen for the treatment of CI due to VILI (Fu et al., 2007; Huang LT. et al., 2014). Another NSAID, parecoxib, was observed the attenuating effect of lung injury in a rat model of VILI as demonstrated by the reduction of local and systemic inflammation (Meng et al., 2017). Interestingly, combined data showed that the use of parecoxib reduced hippocampal inflammation through reducing the expression of PGE2 and COX-2 in the hippocampus and also might have an impact on the elevation of synaptophysin, to play a role in anti-anxiety and memory-enhancing effects (Li et al., 2016; Wang et al., 2017). These results suggest that parecoxib has great potential to treat VILI-induced impairment of cognitive function. There are also emerging treatments that can improve VILI through reversing inflammation. For example, the anti-inflammatory effects of inhaled hydrogen sulfide during mechanical ventilation, either pre-treatment or post-treatment, and its mechanism may be related to thermoregulation and the inhibitory effects of cytokine release and reactive oxygen cluster formation (Faller et al., 2010; Faller et al., 2017). Other studies have found that treating rats with mesenchymal stem cells for VILI prior to mechanical ventilation not only reduces the inflammatory response in the lungs but also enhances lung recovery after VILI (Curley et al., 2012; Curley et al., 2013; Lai et al., 2015). What makes them potentially more promising in treating VILI-induced cognitive impairment is the fact that they have a protective effect on the hippocampal neurons (Tfilin et al., 2010; Li et al., 2011). In summary, a variety of medications that have the effect of eliminating the ventilator-induced inflammatory response have been extensively studied, but most are limited to preclinical animal studies not yet been clinically validated.

### 4.3 Adjunctive interventions

Most of the above two types of interventions are relevant to the basic treatment of patients with ventilator-induced lung injury, and the following, although literally an adjunctive function, is also an important guide to the treatment of cognitive impairment due to VILI. The field of research on non-pharmacological interventions has gradually expanded and can be loosely categorized as the development of physical interventions to cognitive interventions (Brummel et al., 2014), both of which are discussed below.

#### 4.3.1 Physical interventions

A prospective cohort study demonstrated that physical intervention in mechanically ventilated patients promoted walking and concluded that it was safe and feasible (Bailey et al., 2007). Currently, it has been proven that early mobilization after mechanical ventilation may reduce the adverse effects of ventilation, including long-term cognitive impairment (Patel et al., 2023). Schweickert conducted a randomized controlled trial with 104 mechanically ventilated patients undergoing early exercise and mobilization and they presented improved functional status and cognitive outcomes (duration of delirium) at discharge compared to the control group (Schweickert et al., 2009). Other studies have also shown that early mobilization reduces the incidence of delirium and ventilator-associated pneumonia in mechanically ventilated patients in the ICU (Hodgson et al., 2016; Wang et al., 2020). In addition to the means described above, prone position, several preclinical studies have shown that its mechanism may include an even and homogenous distribution of pulmonary strain and a decline in apoptosis in pulmonary tissue in terms of mitigating VILI (Valenza et al., 2005; Nakos et al., 2006). Apart from this, a systematic review of ten trials concluded that prone positioning reduces the risk of pneumonia and improves oxygenation in mechanically ventilated patients (Sud et al., 2008). Furthermore, in a C57BL/6J mice VILI model, Sparrow et al. initially reported that prone positioning diminished inflammatory cytokines and neuronal damage in the frontal brain and hippocampus via the IL-6 trans-signaling pathway, which might assist with CI due to VILI (Sparrow et al., 2022). These suggest that the therapeutic effect of postural therapy for VILI itself and the resulting cognitive impairment has also been validated.

#### 4.3.2 Cognitive interventions

The role of non-invasive brain stimulation strategies for cognitive functioning is gradually gaining attention from researchers (Vicario et al., 2019). Turon et al. found that a cognitive intervention strategy on virtual reality technology was safe and effective in stimulating cognitive functions in the brains of mechanically ventilated patients (Turon et al., 2017). A study in rats with mechanical ventilation-induced brain impairment found that rhythmic nasal air-puffing improved memory function and modulated oscillatory activity in the cognitive function area of the brain in the intervention group (Ghazvineh et al., 2021). There are some novel studies that confirm the feasibility of music interventions for mechanically ventilated patients and show that music interventions can improve patients’ delirium and distress levels (Khan et al., 2020; Dallı Ö et al., 2023), but more research is needed to determine the mechanisms by which music promotes cognitive function.

### 4.4 Individualized therapy

Previously described intervention strategies for the prevention and treatment of cognitive impairment due to VILI may have variable effects due to different patient disease progression, patient pathophysiological characteristics, or environmental factors, and thus improved individualization of treatment based on them may protect cognitive function in patients with VILI in a relatively more comprehensive manner. An observational study found that assessing pain, sedation, and delirium levels in ICU patients and managing individualized titration of analgesics, sedatives, and antipsychotics on this basis improved clinical outcomes such as duration of mechanical ventilation and medically induced coma (Skrobik et al., 2010). ABCDEF bundle is a patient-focused, symptom-based, individualized treatment strategy that focuses on effective tools to monitor symptoms of delirium, distress, and agitation and develops six patient-based care approaches to improve functional and cognitive outcomes (Ely, 2017). A study that included 15,226 ICU adults confirmed that the ABCDEF bundle improves clinical outcomes such as coma, delirium, and duration of mechanical ventilation in mechanically ventilated patients (Pun et al., 2019). These individualized interventions demonstrate the potential for treating cognitive impairment due to VILI and it is hoped that more protocols and improved protocols will emerge in the future.

## 5 Conclusion

Over the past few years, VILI has become a research hotspot due to being a risk factor for cognitive impairment during mechanical ventilation. In this review, we focused on the pathological process and analyzed the molecular mechanism of cognitive impairment induction. Mechanical ventilation achieves neuronal damage by inducing hypoxemia and inflammatory response of CNS. At the same time, VILI can also disrupt CNS homeostasis through direct neural pathways and by altering the release of neurotransmitters. In order to reduce the organ destruction caused by VILI itself and improve the prognosis of patients with cognitive impairment, we summarized the combination of LPVS and pharmacologic therapy with adjunctive interventions to provide patients with individualized treatment references. In conclusion, VILI contributes greatly to the development of cognitive impairment, and the precise formulation of ventilation parameters as well as optimal drug therapy helps to improve prognosis. Rapidly advancing basic science research may better characterize VILI’s role in the development of cognitive impairment.
